# Emphysematous Pyelonephritis Complicated With Hyperglycemic Hyperosmolar State and Sepsis: A Case Report and Literature Review

**DOI:** 10.7759/cureus.25498

**Published:** 2022-05-30

**Authors:** Daniel A Fernandez Felix, Gloriana Madrigal Loria, Sapna Sharma, Mahmoud Ali, Carlos E Arias Morales

**Affiliations:** 1 Medicine, SBH (St. Barnabas Hospital) Health System, New York City, USA; 2 Clinical Medicine, City University of New York School of Medicine, New York City, USA

**Keywords:** hyperglycemic hyperosmolar syndrome, hyperglycemic hyperosmolar nonketotic state, hyperglycemic hyperosmolar state, sepsis, shock, emphysematous pyelonephritis

## Abstract

Emphysematous pyelonephritis (EPN) is an acute life-threatening necrotizing infection of the renal parenchyma and perirenal tissues. There are multiple treatment strategies for EPN depending on the initial classification; over the last three decades, the treatment approach has favored kidney sparing strategies and the use of nephrectomy only as salvage therapy. We report a case involving a patient with unilateral emphysematous pyelonephritis complicated with hyperglycemic hyperosmolar state (HHS), sepsis, and multiple risk factors associated with poor prognosis who was successfully treated with conservative management sparing nephrectomy. This case report aims to create awareness among clinicians that even in the presence of multiple risk factors for poor prognosis, conservative management should be considered before nephrectomy.

## Introduction

Emphysematous pyelonephritis (EPN) is an acute life-threatening necrotizing infection of the renal parenchyma and perirenal tissues [1]. Diabetes mellitus, female gender, and urinary tract obstruction are the major risk factors for the development of EPN [2,3]. Class 3a EPN, complicated with hyperglycemic hyperosmolar state (HHS) and sepsis, is an aggressive and severe form of EPN that, if not recognized in a timely manner and properly treated, can progress rapidly, leading to death [4,5]. We report a case involving a patient with unilateral emphysematous pyelonephritis complicated with HHS, sepsis, and multiple risk factors associated with poor prognosis who was successfully treated with conservative management sparing nephrectomy.

## Case presentation

A 65-year-old male with a past medical history of type 2 diabetes mellitus with last HbA1C of 17% (Reference range: 4-5.5%) treated with basal insulin regimen and a previous stroke without residual neurological deficit presented to the emergency room (ER) with the chief complaint of lightheadedness. The patient reported generalized weakness, lightheadedness, and dysuria for several days. He was not a reliable historian and no family members were available to provide further information. On the physical exam, the patient was oriented to person only, tachycardic, and showed right costovertebral angle tenderness.

Initial blood tests (Table 1) showed elevated white blood cell (WBC) count, thrombocytopenia, hyponatremia, low serum carbon dioxide, elevated serum glucose, elevated serum creatinine, high blood osmolality, elevated anion gap, small presence of serum ketones and lactic acidosis, and elevated procalcitonin. Urinalysis was remarkable for glucosuria, ketonuria, hematuria, positive leukocyte esterase, bacteriuria, and elevated WBCs. The initial venous blood gas was remarkable for low pH as described in Table 2.

**Table 1:** Initial blood tests

Test name	Result	Reference range
White blood cells	17.1 x 109/L	4.2-9.1 x 10*3/uL
Platelet count	120 x 109/L	150-450 x 10*3/uL
Serum glucose	1092 mg/dL	70-99 mg/dl
Serum creatinine	3.7 mg/dL	0.6-1.2 mg/dl
Serum sodium	116 mEq/L	135-145 mEq/L
Serum carbon dioxide	17 mEq/L	23-30 mEq/L
Blood osmolarity	315 mosm/kg	290-300 mosm/kg
Serum ketones	Small	Negative
Anion gap	23 mEq/L	7-16 mEq/L
Lactic acid	5.0 mmol/L	0.5-2.2 mmol/L

**Table 2:** Initial venous blood gas PCO2: partial pressure of carbon dioxide

Venous blood gas		
pH	7.29 pH units	Reference: 7.3-7.4 pH units
PCO2	40 mmHg	Reference: 42-48 mmHg
Bicarbonate	18 mEq/L	Reference: 24-30 mEq/L

**Figure 1:**
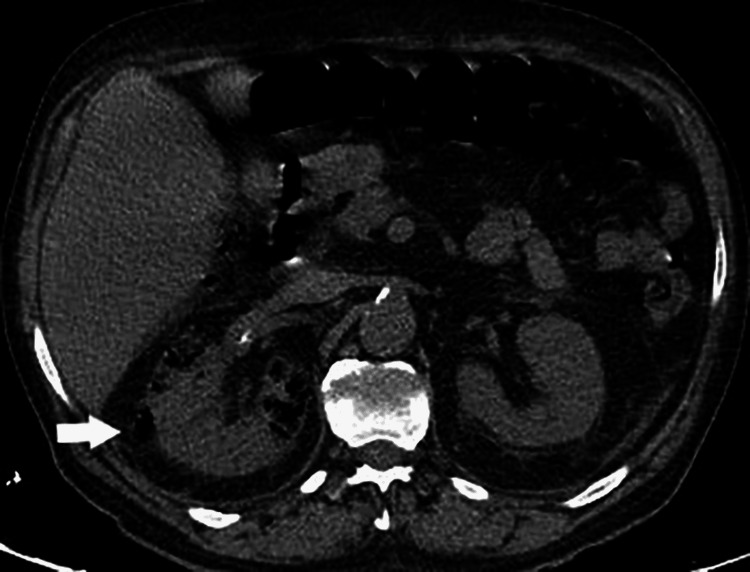
Right EPN Day 1: Axial non-contrast view showing collections of air identified throughout the renal parenchyma on the right. Day one of hospital admission. EPN: emphysematous pyelonephritis

**Figure 2:**
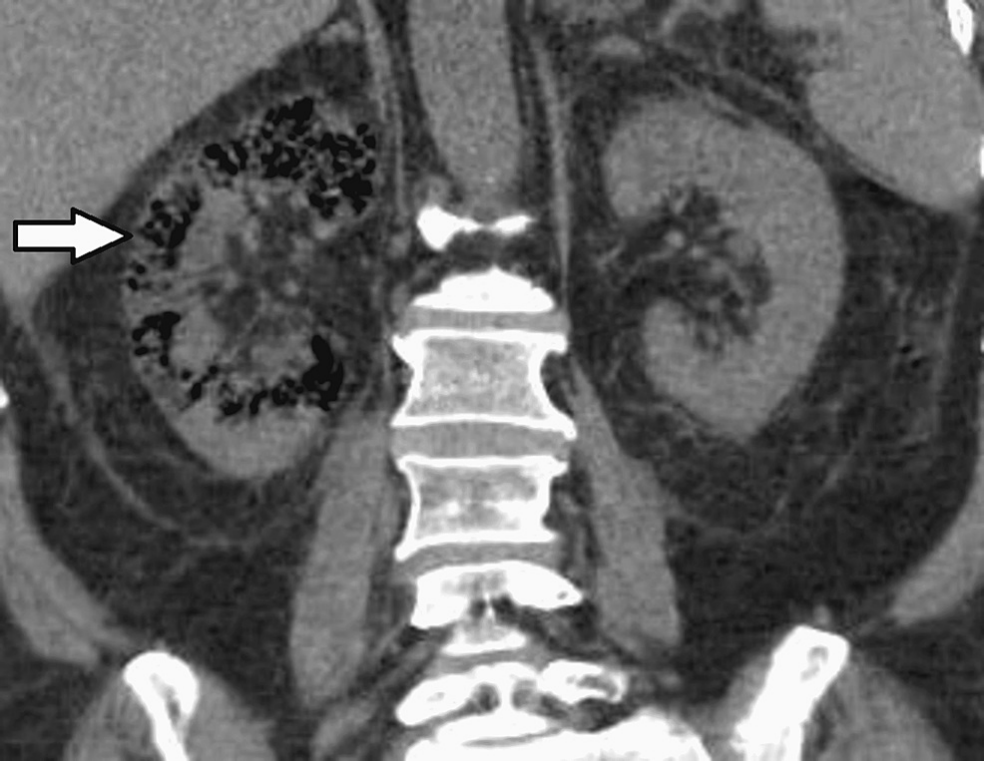
Right EPN Day 1 : Coronal non-contrast view showing collections of air identified throughout the renal parenchyma on the right. Day one of hospital admission. EPN: emphysematous pyelonephritis

Ultrasound (USG) and computed tomography (CT) scans of the abdomen were performed showing multiple increased echogenicity involving the right kidney and collections of air identified throughout the right renal parenchyma, respectively (Figures 1, 2). Based on the results of the initial workup, a diagnosis of EPN complicated with HHS and sepsis was made.

The patient was admitted to the medical intensive care unit, and treatment was started with aggressive intravenous (IV) fluids, empiric antibiotics (vancomycin, meropenem, and ciprofloxacin), and insulin infusion as per protocol. The anion gap closed 30 hours after the insulin was started. The patient initially required vasopressors due to hypotension. A decision for conservative treatment with antibiotics and management of HHS was made by a multidisciplinary team (critical care, infectious disease, urology, and interventional radiology) since no drainable fluid collections were appreciated on radiological imaging. 

**Figure 3:**
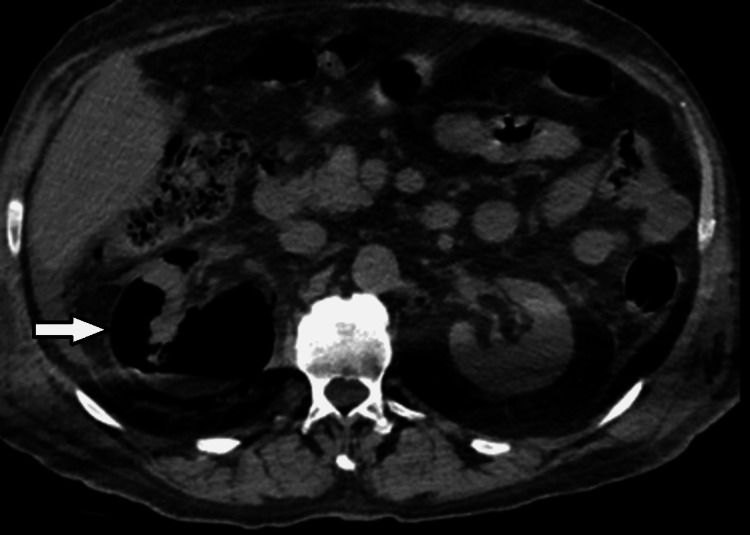
Right EPN Day 3: Axial non-contrast view showing interval worsening in right renal emphysematous pyelonephritis with increased air throughout the kidney. Day three of hospital admission. EPN: emphysematous pyelonephritis

**Figure 4:**
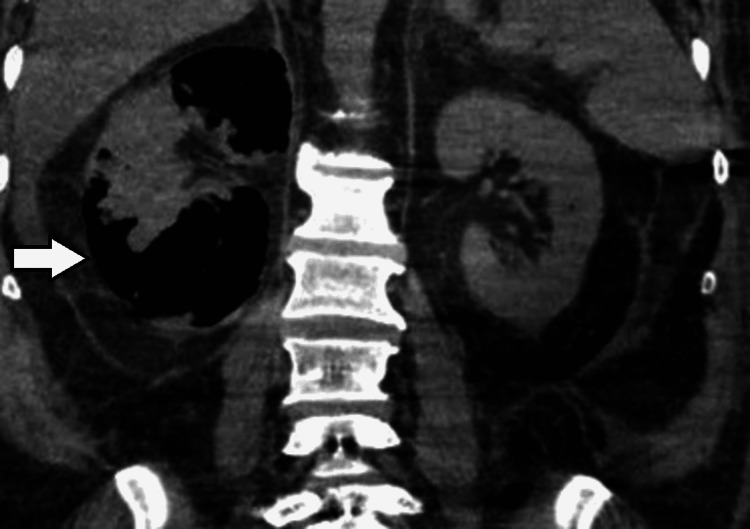
Right EPN Day 3: Coronal non-contrast view showing interval worsening in right renal emphysematous pyelonephritis with increased air throughout the kidney. Day three of hospital admission. EPN: emphysematous pyelonephritis

On day three, a repeated abdominal CT scan showed increased air throughout the kidney (Figures 3, 4), associated with a rise in WBCs and severe thrombocytopenia (37× 109 /L); however, as the patient was off vasopressor support and improving clinically, a decision was made to continue conservative management.

**Figure 5:**
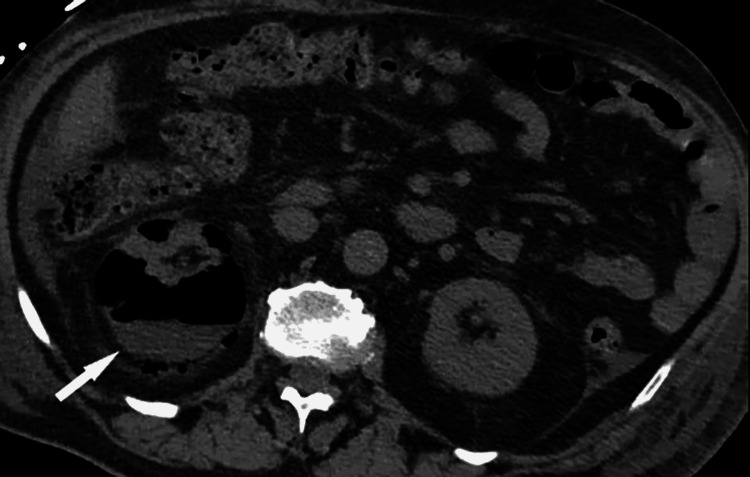
Right EPN Day 5: Axial view non-contrast showing fluid/gas level with fluid collection in the perinephric area in the setting of EPN consistent with abscess formation. Day five of hospital admission. EPN: emphysematous pyelonephritis

**Figure 6:**
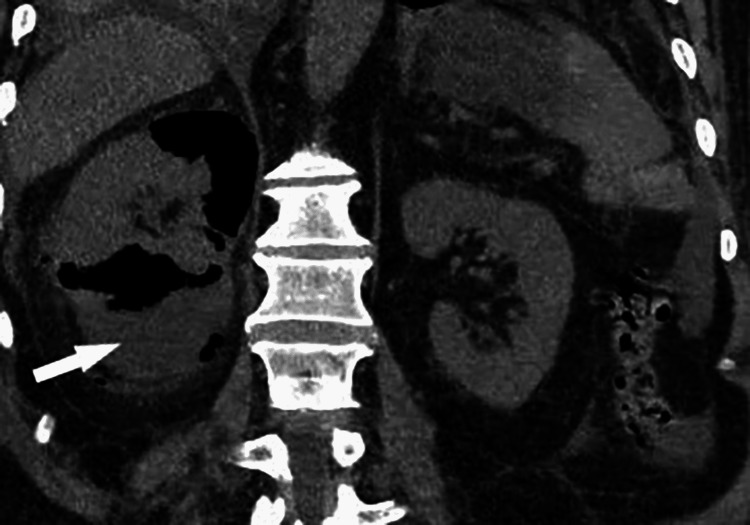
Right EPN Day 5: Coronal view non-contrast showing fluid/gas level with fluid collection in the perinephric area in the setting of EPN consistent with abscess formation. Day five of hospital admission. EPN: emphysematous pyelonephritis

**Figure 7:**
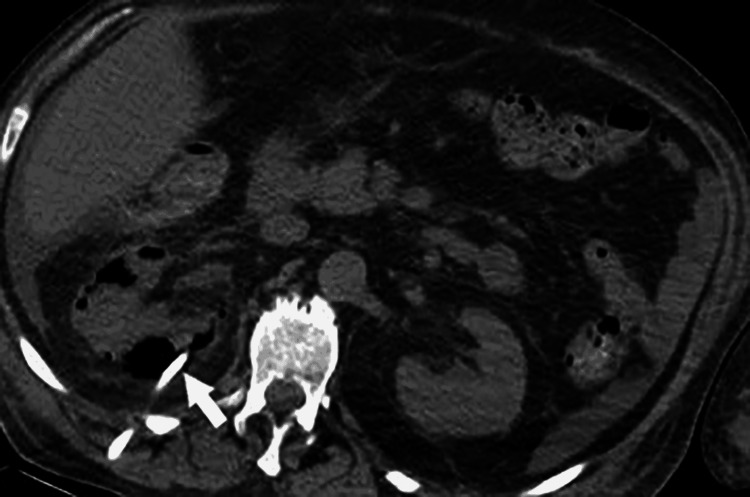
Axial non-contrast view showing percutaneous catheter draining the previously noted right renal abscess. EPN persisted on day six of hospital admission. EPN: emphysematous pyelonephritis

**Figure 8:**
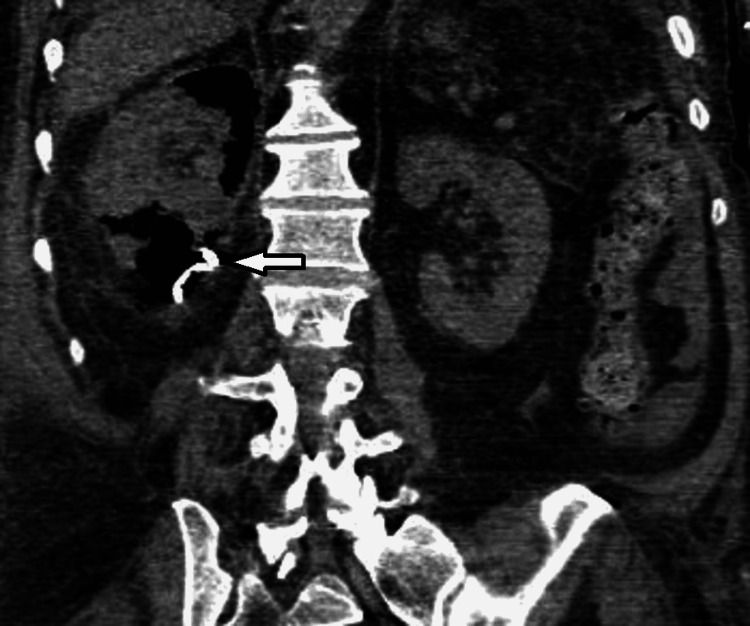
Coronal non-contrast view showing percutaneous catheter draining the previously noted right renal abscess. EPN persisted on day six of hospital admission. EPN: emphysematous pyelonephritis

On day five, a repeat abdominal CT showed fluid/gas level with fluid collection in the perinephric area (Figures 5, 6) consistent with abscess. A 10-french CT-guided drainage tube was placed on the right renal emphysematous abscess by interventional radiology (Figures 7, 8), draining bloody, odorless fluid. Urine cultures obtained grew *Candida albicans* and *Escherichia coli* and cultures from renal abscess fluid grew *E. coli*. Antibiotics were narrowed according to their respective sensitivities.

By day 16 of hospitalization, inflammatory markers, WBC, and serum creatinine trended down, and platelets normalized. The patient was hemodynamically stable and asymptomatic with normal mental status. Subsequently, the drain was removed and the patient decided to leave against medical advice declining to complete treatment with intravenous antibiotics. The patient was discharged on oral antibiotics with outpatient follow-up with urology and primary medical doctor; however, unfortunately, the patient was then lost to follow-up.

## Discussion

The initial presentation of the diabetic 65-year-old man with altered mental status, hypotension, severe hyperglycemia, and high serum osmolarity are consistent with HHS [6]. Small ketones in the serum can be seen in HHS. The elevated anion gap can be explained by lactic acidosis and acute kidney injury (AKI). This indicates this patient presented with EPN in the setting of severe sepsis secondary to EPN. 

The most common risk factors for developing EPN are diabetes mellitus, female gender, and urinary tract obstruction [2]. *E coli* and *Klebsiella pneumoniae* are the most common etiologic agents identified in EPN [2,7]. Multiple mechanisms for gas production within the renal parenchyma have been described. Fermentation of glucose by glucose-fermenting bacteria [8], fermentation products from tissue necrosis producing carbon dioxide [9], and impaired transportation of gas produced by rapid catabolism leads to gas accumulation in the tissues [10]. Histopathology findings were Kimmelstiel-Wilson nodules, foci of infarction (glomeruli and tubules), necrotizing vasculitis, vascular thrombosis, multiple abscesses, and gas-filled spaces surrounded by inflammatory cells [2,11,12]. The clinical presentation of EPN is similar to those seen in severe, acute pyelonephritis characterized by fever, chills, flank or abdominal pain, nausea, and vomiting [13,14]. An abdominal CT scan is the most sensitive and specific test to diagnose EPN [2,15]. In 1996, two types of EPN were described by Wan et al. with prognostic implications [12]. Type 1, also known as "dry type," is characterized by the absence of fluid collection on CT scan abdomen and type 2, also known as "wet type," is characterized by fluid collections with bubbly or loculated gas or gas in the collecting system. According to Wan et al., type 1 EPN mortality was 69% compared to 18% for type 2. In 2000, Huang and Tseng classified EPN into four classes according to radiological CT scan findings; class 1 was defined as gas only in the collecting system, class 2 was defined as gas in the renal parenchyma without extension to extrarenal space, class 3 is subdivided into class 3A, defined as an extension of gas or abscess to perinephric space, and class 3B, defined as an extension of gas or abscess to pararenal space, and class 4 was defined as bilateral EPN or solitary kidney with EPN [2].

The accepted treatment for EPN in the 1980s was emergency nephrectomy, open surgical drainage, or both plus antibiotics, with a reported mortality rate of 40-50% [16]. The current treatment strategies include medical management (MM) alone, MM plus percutaneous catheter drainage (PCD), MM plus PCD plus double J stents, MM plus nephrectomy, and MM plus PCD plus emergency nephrectomy. These treatment strategies have shown lower mortality rates compared to the 1980s approach [2,7,17,18]. Over the last three decades, the treatment approach has favored kidney sparing strategies and the use of nephrectomy only as salvage therapy; however, multiple predictive factors for mortality have been identified, which can orient the medical team on which treatment strategy should be followed, expecting better outcomes. In a case series of 39 patients, severe hyponatremia, thrombocytopenia, altered mental status, and renal failure at presentation were associated significantly with increased mortality [3]. A meta-analysis done by Falagas concluded that type 1 EPN, bilateral EPN, concomitant thrombocytopenia, conservative treatment, creatinine above 2.5 mg/dL, and systolic blood pressure less than 90 mmHg were associated with increased mortality [19]. Huang and Tseng's approach to management is based on staging and risk factors for poor prognosis. They recommended conservative management (MM +/- PCD) of EPN stage 1, stage 2, and stages 3-4 with less than two risk factors (thrombocytopenia, acute renal failure, disturbance of consciousness, and shock), and in patients with two or more risk factors, they stated nephrectomy should be promptly attempted [2].

In our case, most of the risk factors associated with poor prognosis were present, including altered mental status, shock, thrombocytopenia, renal failure, and hyponatremia. Our patient could have been considered for early nephrectomy. However, a kidney sparing strategy was chosen with favorable results, questioning what other variables should be considered before proceeding with a nephrectomy. The patient developed a new intrarenal abscess followed by a sudden rise in WBCs and down-trending platelets; we should always suspect a complicated EPN with an abscess in this setting and consider repeating an abdominal CT scan.

## Conclusions

This case report aimed to support the current treatment options for EPN currently described in the literature. In addition, we intend to create awareness among clinicians about the disease and that even in the presence of multiple risk factors for poor prognosis, conservative management for EPN should be considered before nephrectomy.
